# Repression of the Glucocorticoid Receptor Aggravates Acute Ischemic Brain Injuries in Adult Mice

**DOI:** 10.3390/ijms19082428

**Published:** 2018-08-17

**Authors:** Yong Li, Lei Huang, Qingyi Ma, Katherine R. Concepcion, Minwoo A. Song, Peng Zhang, Yingjie Fu, Daliao Xiao, Lubo Zhang

**Affiliations:** The Lawrence D. Longo, MD Center for Perinatal Biology, Department of Basic Sciences, Loma Linda University, School of Medicine, Loma Linda, CA 92350, USA; yoli@llu.edu (Y.L.); lehuang@llu.edu (L.H.); qma@llu.edu (Q.M.); kconcepcion@llu.edu (K.R.C.); minsong@llu.edu (M.A.S.); pzhang@llu.edu (P.Z.); psgina4628@gmail.com (Y.F.); dxiao@llu.edu (D.X.)

**Keywords:** GR, MR, GR siRNA, ischemic stroke, MCAO, inflammation, BDNF/TrkB

## Abstract

Strokes are one of the leading causes of mortality and chronic morbidity in the world, yet with only limited successful interventions available at present. Our previous studies revealed the potential role of the glucocorticoid receptor (GR) in the pathogenesis of neonatal hypoxic-ischemic encephalopathy (HIE). In the present study, we investigate the effect of GR knockdown on acute ischemic brain injuries in a model of focal cerebral ischemia induced by middle cerebral artery occlusion (MCAO) in adult male CD1 mice. GR siRNAs and the negative control were administered via intracerebroventricular (i.c.v.) injection 48 h prior to MCAO. The cerebral infarction volume and neurobehavioral deficits were determined 48 h after MCAO. RT-qPCR was employed to assess the inflammation-related gene expression profiles in the brain before and after MCAO. Western Blotting was used to evaluate the expression levels of GR, the mineralocorticoid receptor (MR) and the brain-derived neurotrophic factor/tropomyosin receptor kinase B (BDNF/TrkB) signaling. The siRNAs treatment decreased GR, but not MR, protein expression, and significantly enhanced expression levels of pro-inflammatory cytokines (IL-6, IL-1β, and TNF-α) in the brain. Of interest, GR knockdown suppressed BDNF/TrkB signaling in adult mice brains. Importantly, GR siRNA pretreatment significantly increased the infarction size and exacerbated the neurobehavioral deficits induced by MCAO in comparison to the control group. Thus, the present study demonstrates the important role of GR in the regulation of the inflammatory responses and neurotrophic BDNF/TrkB signaling pathway in acute ischemic brain injuries in adult mice, revealing a new insight into the pathogenesis and therapeutic potential in acute ischemic strokes.

## 1. Introduction

Strokes rank as the fifth cause of mortality and the leading cause of major disability in the United States [1,2]. Approximately about 795,000 stroke events occur in the United States annually, of which more than 80% cases are ischemic attacks [1]. Every 4 min, someone will die from a stroke attack [1]. The total direct medical stroke-related costs were approximately $71.6 billion in 2012 and such costs will be tripled to $184.1 billion in 2030 [1,3]. However, the available clinically practicable interventions are still very limited after decades of practice and investigation, which is at least in part attributable to the incomplete understanding of its basic pathogenesis. Therefore, it is urgent to further explore the potential mechanisms underpinning acute stroke events and to develop feasible and effective intervention strategies. 

Growing evidence has shown the critical role of glucocorticoids in the brain’s growth and development. Endogenous or exogenous glucocorticoids exert significant effects on the brain’s activities in various physiological and pathological conditions chiefly via the activation of the glucocorticoid receptor (GR) [4]. Aberrant GR activity is implicated in diverse brain pathologies [4]. Our previous studies revealed that GR activation in the developing rat brain confers neuroprotective effects in the model of neonatal HIE [5,6]. In addition, maternal hypoxia induced the down-regulation of GR in immature brain programs an ischemic-sensitive phenotype in neonatal rat pups [7]. However, the role of GR signaling in the fully developed and mature brain with ischemic challenges is still vague and needs to be further elucidated.

In the present study, we explored the potential role of GR in the setting of acute ischemic brain injuries with a classic middle cerebral artery occlusion (MCAO) model in adult mice. Herein, we present evidence that the repression of basal GR in the adult mouse brain induces heightened pro-inflammatory responses and suppresses neurotrophic BDNF/TrkB signaling, which contributes to an enhanced infarct size and worsened neurobehavioral deficits induced by MCAO insult. The findings provide new insights into a potential intervention strategy of targeting GR in the management of acute ischemic stroke events in clinics. 

## 2. Results

### 2.1. GR siRNA Treatment Repressed GR Expression in the Brain

**Figure 1:**
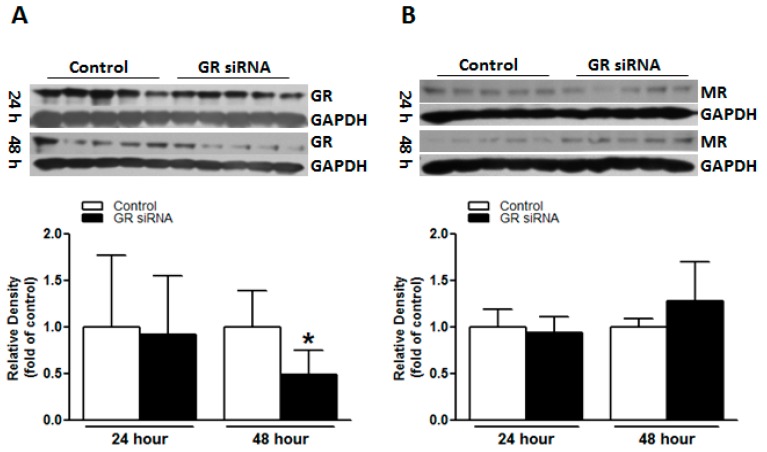
The glucocorticoid receptor (GR) siRNA repressed GR expression in mice brain. The mice received either GR siRNA (100 pmol) or its negative control (Neg. Control; 100 pmol) via intracerebroventricular (i.c.v.) injection. The brain samples were collected at 24 h and 48 h after i.c.v. treatment. Glucocorticoid receptor (GR) protein abundance (**A**) and mineralocorticoid receptor (MR) protein abundance (**B**) were determined via western blotting at 24 h and 48 h, respectively. *n* = 5. * *p* < 0.05, GR siRNA vs. Control.

GR siRNAs (100 pmol) were administered via i.c.v. injection, and GR protein expression in the brain was determined 48 h after the treatment. As demonstrated in Figure 1A, GR siRNA treatment significantly down-regulated GR protein abundance in the mouse brain at 48 h after injection, whilst no marked effects at 24 h. Given the potentially complex balance effect of GR and MR in the brain [8,9,10,11,12], we also investigated the possible effects of GR down-regulation on the expression level of MR. Western blotting indicated that MR protein abundance in the brain was not significantly altered by the GR siRNA treatment (Figure 1B). 

### 2.2. GR Repression Induced Heightened Inflammatory Responses in the Mouse Brain

**Figure 2:**
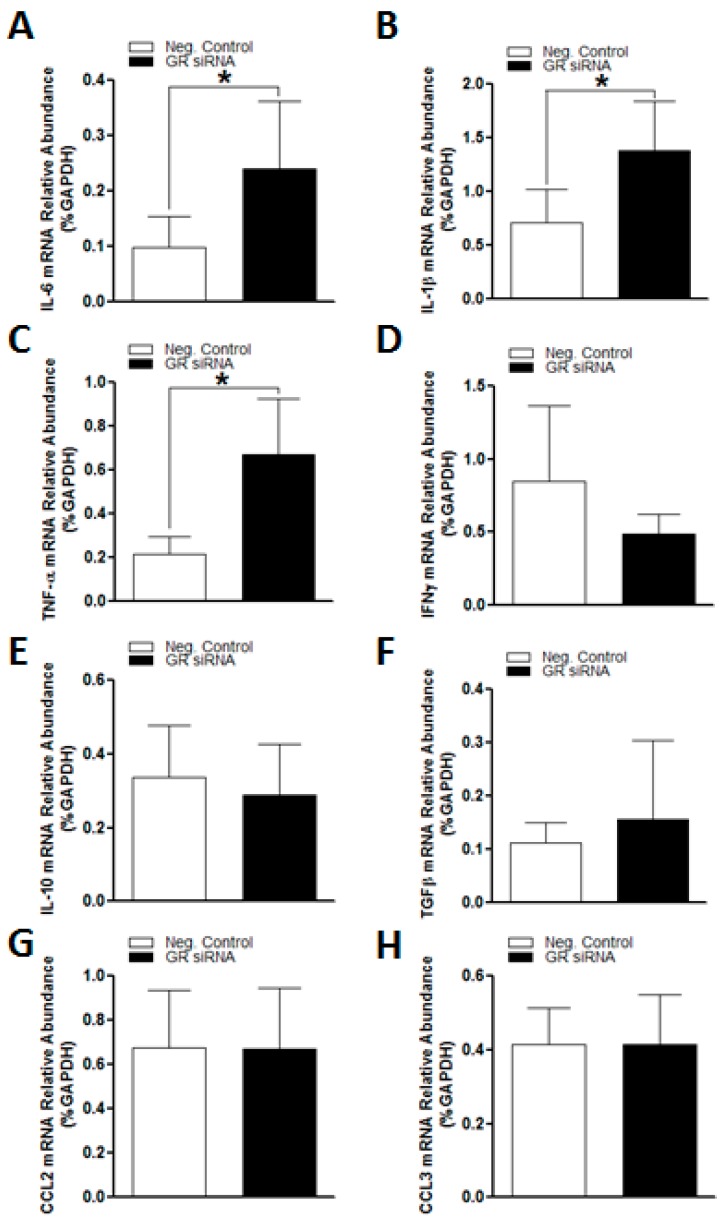
The GR repression increased expression of pro-inflammatory genes in mice brains before MCAO treatment. The mice received either GR siRNA (100 pmol) or its negative control (Neg. Control; 100 pmol) via intracerebroventricular (i.c.v.) injection. The brain samples were collected at 48 h after i.c.v. injection. No middle cerebral artery occlusion (MCAO) was conducted. Quantitative reverse transcription polymerase chain reaction (RT-qPCR) was utilized to assess inflammation-related genes expression profiles, including IL-6 (**A**), LI-1β (**B**), TNF-α (**C**), IFN-γ (**D**), IL-10 (**E**), TGF-β (**F**), CCL2 (**G**) and CCL3 (**H**), respectively. *n* = 5. * *p* < 0.05, GR siRNA vs. Neg. Control.

Given the pivotal role of GR in the modulation of inflammatory responses, we examined the expression profiles of key inflammation-related genes in the mouse brain via RT-qPCR. As shown in Figure 2, the GR knockdown by GR siRNAs markedly increased expression levels of pro-inflammatory cytokines, including IL-6, IL-1β, and TNF-α (Figure 2A–C), suggesting a potential role of endogenous glucocorticoids activating the GR and repressing pro-inflammatory response in the mouse brain. Of interest, GR knockdown did not exert effects on expression levels of anti-inflammatory genes—IL-10 and TGF-β (Figure 2E,F)—and inflammatory chemokines—CCL2 and CCL3 (Figure 2G,H). 

**Figure 3:**
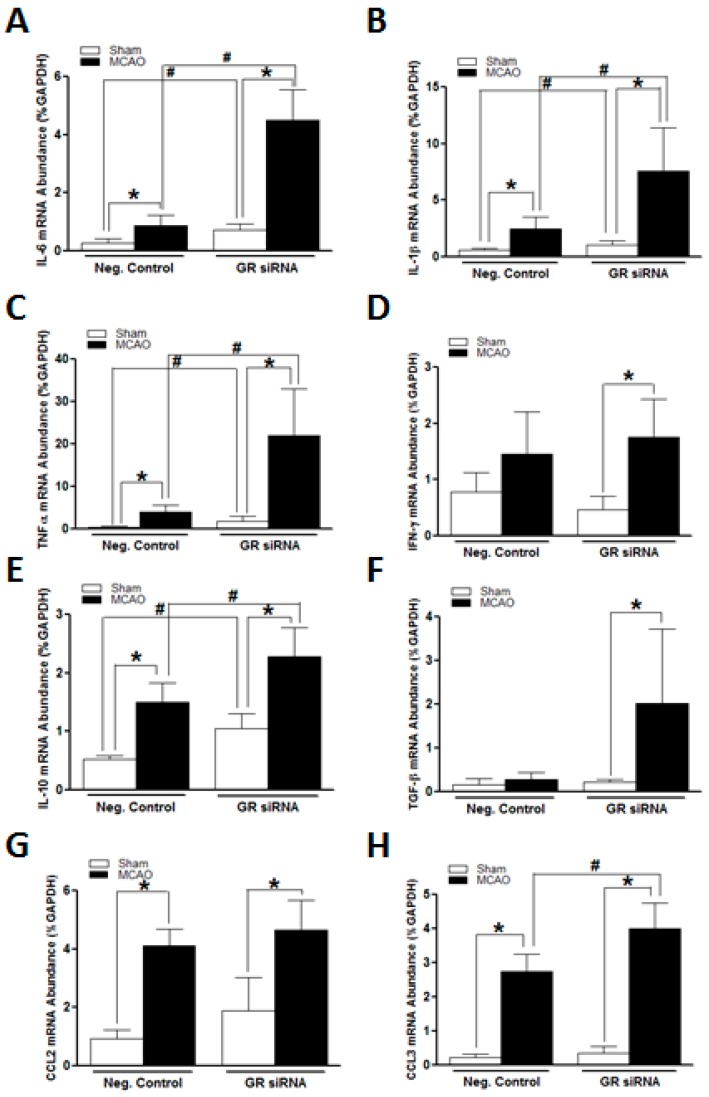
The GR repression induced heightened inflammatory responses in mice brains following MCAO. The mice received either GR siRNA (100 pmol) or its negative control (Neg. Control; 100 pmol) via intracerebroventricular (i.c.v.) injection. A middle cerebral artery occlusion (MCAO) was performed 48 h after i.c.v. injection. Sham animals were subjected to left internal carotid artery exposure but without suture insertion. The brain samples were collected at 12 h after MCAO induction. A quantitative reverse transcription polymerase chain reaction (RT-qPCR) was utilized to assess inflammation-related genes expression profiles, including IL-6 (**A**), LI-1β (**B**), TNF-α (**C**), IFN-γ (**D**), IL-10 (**E**), TGF-β (**F**), CCL2 (**G**) and CCL3 (**H**), respectively. *n* = 5. * *p* < 0.05, MCAO vs. sham; # *p* < 0.05, GR siRNA vs. Neg. Control.

We further assessed the expression profiles of inflammation-related genes in adult mice brains subjected to the MCAO challenge. As shown in Figure 3, the MCAO challenge itself induced a significant up-regulation of pro- and anti-inflammatory genes in the brains of both control and treatment animals, including IL-6, IL-1β, TNF-α, IL-10, CCL2, and CCL3 (Figure 3A–C,E,G,H), indicating a pivotal role of neuroinflammation in the pathology of acute ischemic stroke events. Interestingly, MCAO insult induced a significant increase in IFN-γ and TGF-β only in the GR siRNA treated animals (Figure 3D,F). Of importance, in comparison to the control group, the GR siRNA treated animals demonstrated much higher increased levels of pro-inflammatory genes, IL-6, IL-1β, and TNF-α (Figure 3A–C), at 12 h after the MCAO induction, which is consistent with the profile patterns without MCAO insult. We also observed a significant increase in the IL-10 and CCL3 genes in GR siRNA treated mice brain compared with the controls (Figure 3E,H). 

### 2.3. GR Repression Suppressed BDNF/TrkB Signaling in Mice Brains

**Figure 4:**
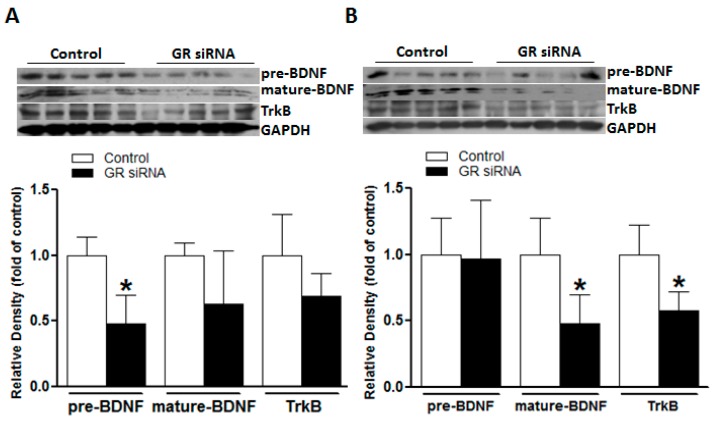
The GR repression inhibited brain-derived neurotrophic factor/tropomyosin receptor kinase B (BDNF/TrkB) signaling in mice brains. The mice received either GR siRNA (100 pmol) or its negative control (Neg. Control; 100 pmol) via intracerebroventricular (i.c.v.) injection. The brain samples were collected at 24 h and 48 h after i.c.v. treatment. The brain-derived neurotrophic factor (BDNF; pre- and mature-BDNF) and Tropomyosin receptor kinase B (TrkB) were determined via western blotting at 24 h (**A**) and 48 h (**B**), respectively. *n* = 5. * *p* < 0.05, GR siRNA vs. Control.

The neurotrophic effect of BDNF/TrkB signaling is well recognized in a diverse brain physiology and pathology [13,14,15]. We explored a potential role of GR repression in the regulation of BDNF/TrkB signaling in adult mouse brains. We found that the GR repression with GR siRNA treatment significantly decreased the TrkB and mature-BDNF protein abundance in the brain (Figure 4B), suggesting a potential interaction between the GR and BDNF/TrkB signaling in adult mouse brains. GR siRNA treatment also induced a transient decrease of the pre-BDNF level at 24 h, which was restored at 48 h (Figure 4A,B). 

### 2.4. GR Repression Increased Infarction Size and Worsened Neurobehavioral Deficits in MCAO Insult

**Figure 5:**
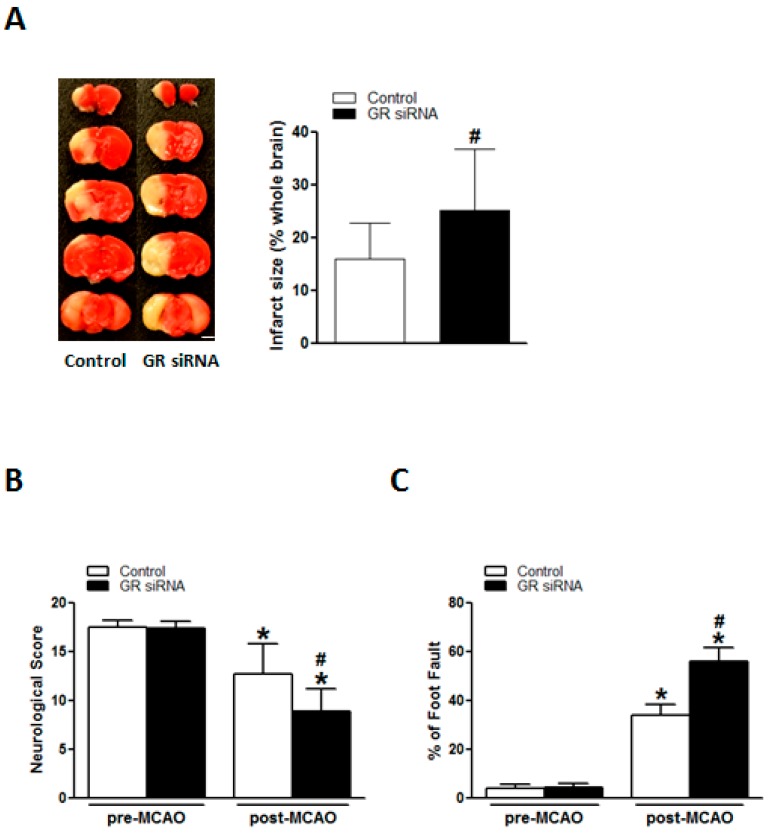
The GR repression increased the infarction size and worsened neurobehavioral deficits in mice with MCAO. The mice received either GR siRNA (100 pmol) or its negative control (Neg. Control; 100 pmol) via intracerebroventricular (i.c.v.) injection. A middle cerebral artery occlusion (MCAO) was performed 48 h after i.c.v. injection. The brain infarction size was assessed 48 h after MCAO conduction (**A**). Neurobehavioral tests including Neurological Score (**B**) and Foot Fault (**C**) were evaluated before MCAO conduction and 48 h after MCAO conduction, respectively. *n* = 10–12. * *p* < 0.05, pre-MCAO vs. post-MCAO; # *p* < 0.05, GR siRNA vs. Control. Scale bar, 2 mm.

Compared to control animals, MCAO induced significantly increased infarction size in the brain of GR siRNA pretreated mice (25.16% ± 11.69% vs. 15.96% ± 6.85%; Figure 5A). Of importance, GR siRNA treated animals presented with a much worse performance in neurobehavioral tests as shown in a decrease in neurological scores (8.88 ± 2.29 vs. 12.75 ± 3.05; Figure 5B) and an increase in percentage values in foot fault test (56.00% ± 5.57% vs. 33.88% ± 4.47%; Figure 5C), suggesting that GR repression in the brain also exacerbates functional outcomes in acute ischemic strokes. 

## 3. Discussion

The present study demonstrated several novel findings: 1. The repression of GR in adult mouse brains induced an enhanced inflammatory response in the context of both physiological and pathological conditions; 2. GR down-regulation inhibited the activity of neurotrophic BDNF/TrkB signaling in adult brains; 3. Of importance, GR repression sensitized the brain to acute ischemic insult, leading to an increased infarct size and worsened neurobehavioral performance in MCAO challenge. Taken together, our present study further revealed a potential role of GR in the homeostasis and pathology of a fully-developed brain.

It is well recognized that the brain is one of the major targets of glucocorticoids [4,5]. Chiefly via the activation of GR, glucocorticoids affect various physiological and pathological processes in the brain, which may be neurodegenerative or neuroprotective, depending on experimental protocols, dosage, time, animal age, strains, and species [4,5,16,17,18,19,20,21,22]. The mineralocorticoid receptor (MR) is another adrenal steroid receptor. GR and MR have different affinities to glucocorticoids, of which MR presents a relatively high affinity and is highly bound at basal levels of glucocorticoids, whilst GR binds glucocorticoids weakly at physiological levels [12]. MR is close to saturation with low basal concentrations of corticosterone, while high corticosterone concentrations in stressful events occupy both MR and GR [12]. MR is mainly found in the limbic areas, particularly the hippocampus, while GR is more widely distributed in all brain regions [12,23]. The dysregulation of MR/GR balance is implicated in various neuropathological conditions [8,9,10]. The potential effect of MR in the regulation of brain ischemic injury has been suggested [6,24,25]. In the present study, we found that the knockdown of GR via GR siRNA i.c.v. exerted no significant influence on MR, providing a model to elucidate the specific effects of GR repression in the pathogenesis of brain ischemia without the compounding influence of MR. 

Inflammation is one of the major pathological processes underpinning various neurodegenerative diseases [26]. At the acute stage of strokes, a vast amount of toxic inflammatory factors secreted by activated brain resided or peripherally infiltrated immune cells synergistically contribute to the irreversible cell death and infarction formation [2,27]. In the present study, we observed a robust up-regulation of various pro- and anti-inflammatory genes, such as IL-6, IL-1β, TNF-α, IL-10, CCL2, and CCL3, in the mouse brain subjected to the MCAO challenge. Of interest, even without an MCAO insult, GR siRNA treated animals demonstrated a significant increase in key pro-inflammatory genes, IL-6, IL-1β, TNF-α, in the brain, indicating that the GR repression impaired normally negative regulation of GR on pro-inflammatory genes and primed the brain in a pre-inflammatory state. This is consistent with the well-documented anti-inflammatory effects of GR [24,28,29]. Of critical importance, GR repression in the brain significantly heightened the inflammatory response induced by the MCAO challenge. There were much higher expression levels of pro-inflammatory factors at 12 h after MCAO in the brains of GR siRNA treated mice compared to the control animals. It is possible that this enhanced inflammatory response sensitizes the brain to the ischemic injury, leading to exacerbated neurological outcomes. Interestingly, the GR siRNA treatment also induced an increase in anti-inflammatory cytokine IL-10 levels after MCAO induction, which may be a compensatory protective response. In the present study, GR repression did not exert effects on the expression of IFN-γ, TGF-β, and CCL2, suggesting its complicated differential regulation effects on inflammation-related factors in adult mouse brains. 

Another novel finding of our study is that basal GR repression significantly inhibited neurotrophic BDNF/TrkB signaling in the brain. BDNF is a member of the neurotrophic family, which promotes neuronal survival, axonal growth, and neuroprotection, and has been recognized as a potential therapeutic agent for different neurodegenerative diseases [30,31,32]. BDNF exerts neuroprotective effects mainly via binding to its high-affinity tropomyosin-receptor kinase B (TrkB) [33,34]. In the present study, accompanied with the down-regulation of basal GR protein, there was a significant decrease of both TrkB and the mature-BDNF protein abundance in the brain, as well as a transient down-regulation of pre-BDNF. These findings indicate that GR repression disturbs the expression pattern of BDNF/TrkB, implying a possible linkage between glucocorticoid-GR signaling and the BDNF/TrkB pathway in the mature brain. Indeed, although lacking in detailed descriptions, a few studies began to report the possible linkage of these two vital signaling pathways in the brain. Alboni and colleagues showed that under the basal condition, mice with impaired GR expression displayed lower levels of BDNF exons IX and IV and decreased CRE(BDNF) binding activity with respect to wild-type (WT) mice in the hippocampus [35]. Pandya and colleagues also reported that acute corticosterone exposure induced an increase in the TrkB proteins levels in primary cortical neurons, which was GR-dependent [36]. Jeanneteau and colleagues demonstrated that dexamethasone promoted TrkB receptor phosphorylation in rat brains and offered neuroprotective effects [15]. Chen and colleagues recently speculated that the GR was upstream of TrkB phosphorylation and the activation of BDNF-mediated signaling [37]. Consistently, our present study also demonstrated that the repression of basal GR inhibited the activities of BDNF/TrkB signaling, providing further evidence of the cross-talk between GR and BDNF/TrkB in the adult brain. Given its well-documented neurotrophic effects, it is likely that the GR repression mediated inhibition of BDNF/TrkB also plays an important role in the enhanced susceptibility to acute cerebral ischemic injury induced by MCAO as observed in the present study. One of our recent studies also revealed that perinatal nicotine exposure induced the down-regulation of BDNF/TrkB, which resulted in an increased infarction size in neonatal rat brain hypoxic and ischemic injuries [38]. Both glucocorticoid-GR and BDNF/TrkB play critical roles in the brain’s physiology and pathology, their potential cross-talk linkages are warranted further exploration.

Consistent with our previous studies in neonatal rat brains, the present study revealed the protective effect of GR in adult mouse brain in response to acute ischemic injury. Our previous studies demonstrated that pre- and post-glucocorticoid treatment conferred neuroprotection in neonatal HIE via GR activation, while maternal hypoxia induced the inhibition of GR programs an ischemic sensitive phenotype in neonatal rat brain [5,6,7,39]. In the present study, we administered the siRNA technique to knockdown local basal GR protein levels in the brain, and employed an adult mice MCAO model to investigate the causal role of GR in acute cerebral ischemia in the fully developed brain. As presented in our study, GR siRNA treated mice showed significantly enhanced infarction sizes and exacerbated neurobehavioral deficits compared with the control animals, which was chiefly attributable to the heightened inflammatory responses and suppression of neurotrophic BDNF/TrkB signaling induced by GR repression. Thus, the present finding provides further evidence that GR serves as a common pivotal component implicated in severe ischemic cerebral injuries in both developing and developed brains, suggesting a promising molecular intervention target in the setting of acute ischemic strokes in the adult. 

Several limitations deserve to be mentioned. Our present study evaluated the negative impacts of GR repression on acute ischemic brain injury. Given that chronic functional deficits are a major disability sequela of stroke, additional studies are needed to explore the roles of GR in the long-term functional outcomes in the future. Although we revealed that GR repression induced heightened inflammatory responses and suppressed BDNF/TrkB in adult mouse brains, the detailed underlying molecular mechanisms remain to be determined. In addition, the potential linkage between the suppressed BDNF/TrkB and heightened inflammatory responses induced by GR repression is another exciting puzzle warranted to be further investigated. Of importance, pre-clinical studies should be performed to assess the potential protective effects of GR activation in adult ischemic brain injuries.

## 4. Materials and Methods 

### 4.1. Experimental Animals

A total of 72 eight-week-old male CD1 mice were purchased from Charles River Laboratories (Portage, MI). The animals were maintained at 20 ± 2 °C and housed in a 12-h light-dark cycle with access to food and water ad libitum. The mice were randomly divided into 2 major groups and several subgroups. The two major groups included (1) negative control (*n* = 35); and (2) GR siRNA administered via intracerebroventricular injection (i.c.v) (*n* = 37). Experimental subgroups were listed as followed: Group 1: Western blotting analysis of protein abundance (*n* = 5 in each group at 24 h and 48 h after i.c.v. treatment, respectively); Group 2: quantitative real-time PCR of inflammation-related genes (*n* = 5 in each group at 48 h after i.c.v. treatment and 12 h post-MCAO, respectively); Group 3: infarction and neurobehavioral function assays (*n* = 10 in negative control, *n* = 12 in the GR siRNA treatment). All the experimental procedures and protocols (IACUC# 8160017, approved on 22 July 2016) were approved by the Institutional Animal Care and Use Committee of Loma Linda University and followed the guidelines by the National Institutes of Health Guide for the Care and Use of Laboratory Animals.

### 4.2. Middle Cerebral Artery Occlusion (MCAO)

Focal cerebral ischemia was induced by intraluminal middle cerebral artery occlusion (MCAO) as described previously [2,40]. Briefly, mice were anesthetized with isoflurane (5% for induction and 2% for maintenance) using a face mask. The left common carotid artery and external carotid artery were exposed through a midline neck incision. A 6-0 nylon monofilament coated with silicon rubber (Doccol) was introduced into the left internal carotid artery through the external carotid stump to occlude the origin of the middle cerebral artery and block blood flow to the striatum and cortex. After 50 min of occlusion, reperfusion was introduced by filament withdrawal, and the incision was sutured. During the whole procedure, the mice body temperature was maintained at 37 ± 0.5 °C. No animal died during the MCAO procedure. The mortality of the follow-up study was 10% in the negative control group and 12% in the GR siRNA treatment group. In total, 81 mice were used in this study.

### 4.3. Intracerebroventricular Injection (i.c.v) 

As previously described [2], the adult male CD1 mice were anesthetized with isoflurane (5% for induction and 2% for maintenance) and placed in a stereotaxic frame (Stoelting Co., Wood Dale, Illinois, IL, USA). The ocular ointment was applied to the eyes to prevent drying, animal heads were wiped with 70% ethanol, and the skin on the head was cut open along the midline. A small burr hole (0.5 mm diameter, F.S.T.) was drilled into the skull (0.3 mm posterior to bregma; 1.0 mm lateral to sagittal suture). GR siRNA (Dharmacon) and the negative control (Dharmacon), were prepared according to the manufacturer’s instructions. Total 100 pmol GR siRNA or the negative control with a total volume of 4 μL, were injected over a 5-min period into the left lateral ventricle (depth: 3.0 mm dorsal) with a 10 μL syringe (Stoelting, Wood Dale, Illinois, IL, USA). The wound was closed with a suture, and the mice were waiting to wake up on a heating pad.

### 4.4. Measurement of Infarction Size

The mice were euthanized 48 h after the MCAO treatment. Coronal slices of the brain (2 mm thick) were cut and immersed in a pre-warmed, 2% solution of 2,3,5-triphenyltetrazolium chloride monohydrate for 10 min at 37 °C followed by fixation with 10% formaldehyde overnight [41]. Both sides of each slice were photographed separately. The infarction area was analyzed by the Image J software (Version 1.40; National Institutes of Health, Bethesda, Maryland, MD, USA), summed for each brain, and expressed as a percentage of the whole brain.

### 4.5. Neurobehavioral Tests

The neurological score test and foot-fault test were utilized to evaluate the functional neurological deficits. Both tests were conducted before and 48 h after MCAO by an observer blinded to the experimental groups. In total, six components constitute the neurological score test, including spontaneous activity, symmetry in the movement of limbs, forepaw outstretching, climbing, body proprioception, and response to vibrissae touch [2,42]. The final score for each animal is the summation of all six individual trial scores. The minimum score is 3 and the maximum is 18. Lower scores represent more severe functional deficits. The foot-fault test assesses the forelimb misplacement on a grid during locomotion. For each animal, the total number of steps and the number of times each forelimb fell below the grid during the performance of 5 min or during a total of 50 steps achieved with one forelimb were recorded, and the percentage of the right forelimb (affected by stroke) foot fault to the total steps was calculated [2,43]. In contrast to the neurological score test, higher percentage values represent more severe deficits. Both tests were videotaped and analyzed by an observer blinded to the experimental groups. 

### 4.6. Western Blotting

Protein abundance was determined by western blotting as previously described [39]. Briefly, the animals were euthanized at the scheduled time point and brain samples were isolated. The brain tissues (left half brain) were homogenized in a radioimmunoprecipitation assay (RIPA) lysis buffer (Santa Cruz Biotechnology, Dallas, Texas, TX, USA) followed by further centrifugation at 4 °C for 20 min at 14,000 g, and the supernatants were collected. Then the samples with equal amounts of protein were loaded onto 10% polyacrylamide gel with 0.1% sodium dodecyl sulfate and separated by electrophoresis at 100 V for 120 min. The proteins were then transferred onto nitrocellulose membranes and probed with primary antibodies against GR (1:1000, Cell Signaling Technology, Danvers, MA), MR (1:500, Abcam, Cambridge, Massachusetts, MA, USA), BDNF (1:1000, Santa Cruz Biotechnology, Santa Cruz, California, CA, USA), and TrkB (1:1000, Santa Cruz Biotechnology, Santa Cruz, California, CA, USA), respectively. After washing, the membranes were incubated with secondary horseradish peroxidase-conjugated antibodies. The proteins were visualized with enhanced chemiluminescence reagents, and blots were exposed to Hyperfilm. The results were quantified with the Kodak electrophoresis analysis system and Kodak ID image analysis software (Kodak, Rochester, New York, NY, USA). The target protein abundance was normalized to the abundance of glyceraldehyde-3-phosphate dehydrogenase (GAPDH) as a protein loading control.

### 4.7. Real-time RT-PCR 

As previously described [2], the total RNA (left half brain) was extracted using the TRIzol reagent (Invitrogen) and subjected to reverse transcription with Superscript III First-Strand Synthesis System (Invitrogen), following the manufacturer’s instructions. The target gene mRNA abundance was determined by real-time PCR using SYBR Green Supermix (Biomake). Real-time RT-PCR was performed in a final volume of 25 μL. The PCR reaction conditions were a 5-min hold at 95 °C, followed by 40 cycles of activation for 15 s at 95 °C and annealing/extending for 60 s at 60 °C. The detected target gene primers were listed as the following: IL-6, 5′-ccacggccttccctacttc-3′(forward) and 5′-tgggagtggtatcctctgtgaa-3′(backward); IL-1β, 5′-gagtgtggatcccaagcaat-3′(forward) and 5′-taccagttggggaactctgc-3′(backward); TNF-α, 5′-cagccgatgggttgtacctt-3′(forward) and 5′-ggcagccttgtcccttga-3′(backward); IFN-γ, 5′-tgctgatgggaggagatgtct-3′(forward) and 5′-tgctgtctggcctgctgtta-3′(backward); IL-10, 5′-gatgccccaggcagagaa-3′(forward) and 5′-cacccagggaattcaaatgc-3′(backward); TGF-β, 5′-ccgcttctgctcccactc-3′(forward) and 5′-ggtacctccccctggctt-3′(backward); CCL2, 5′-aggtgtcccaaagaagctgtag-3′(forward) and 5′-aatgtatgtctggacccattcc-3′(backward); and CCL3, 5′-tggaactgaatgcctgagagt-3′(forward) and 5′-taggagatggagctatgcaggt-3′(backward). The PCR reactions were carried out in triplicate, and threshold cycle numbers were averaged for each sample. The Ct values were normalized relative to glyceraldehyde-3-phosphate dehydrogenase (GAPDH). 

### 4.8. Statistical Analysis

The data are expressed as mean ± standard deviation (SD). D’Agostino-Pearson omnibus test and Shapiro–Wilk test were used for normality test. The data were assessed by one way analysis of variance (ANOVA) followed by Neuman-Keuls post-hoc testing for comparisons of multiple groups or Student’s *t*-test (unpaired, two-tailed) for comparisons between two groups, where appropriate, using the Graph-Pad Prism software (GraphPad Software Version 4, San Diego, California, CA, USA). For all comparisons, *p* < 0.05 indicated statistical significance.

## 5. Conclusions

The present study revealed the vital role of GR in the regulation of inflammation in the adult brain. Inhibition of basal GR induces heightened inflammatory responses and suppression of neurotrophic BDNF/TrkB signaling, resulting in an increased susceptibility to acute ischemic brain injury. GR is an essential component in maintenance of brain homeostasis. Aberrant GR activity is implicated in various complex brain pathologies, including acute cerebral ischemia. The findings provide a new insight into the understanding of pathogenesis in ischemic brain injury, and suggest a possible intervention target in the setting of acute ischemic stroke in the adult.
